# Galectin-3: its role in asthma and potential as an anti-inflammatory target

**DOI:** 10.1186/1465-9921-14-136

**Published:** 2013-12-09

**Authors:** Peng Gao, Jodie L Simpson, Jie Zhang, Peter G Gibson

**Affiliations:** 1 Department of Respiratory and Sleep Medicine, Hunter Medical Research Institute, Newcastle NSW2305, Australia; 2 Priority Research Centre for Asthma and Respiratory Diseases and Hunter Medical Research Institute, The University of Newcastle, Callaghan NSW 2308, Australia; 3 Department of Respiratory Medicine, the Second Affiliated Hospital of Jilin University, Changchun, Jilin, China; 4 Changchun Central Hospital, Changchun, Jilin, China

**Keywords:** Galectin-3, Inflammation, Leukocyte, Airway hyperresponsiveness, Airway remodeling

## Abstract

Galectins constitute an evolutionary conserved family that bind to β-galactosides. Increasing evidence shows that galectins are involved in many fundamental biological processes such as cellular communication, inflammation, differentiation and apoptosis. Changes in galectin-3 (Gal-3) expression are commonly seen in cancer and pre-cancerous conditions, and Gal-3 may be involved in the regulation of diverse cancer cell activities that contribute to tumourigenesis, cancer progression and metastasis. In addition, Gal-3 is a pro-inflammatory regulator in rheumatoid arthritis. Gal-3 has been shown to be involved in many aspects in allergic inflammation, such as eosinophil recruitment, airway remodeling, development of a Th2 phenotype as well as increased expression of inflammatory mediators. In an *in vivo* model it was shown that bronchoalveolar lavage (BAL) fluid from ovalbumin-challenged mice contained significantly higher levels of Gal-3 compared to control mice. The molecular mechanisms of Gal-3 in human asthma have not been fully elucidated. This review will focus on what is known about the Gal-3 and its role in the pathophysiological mechanisms of asthma to evaluate the potential of Gal-3 as a biomarker and therapeutic target of asthma.

## Introduction

**Figure 1:**
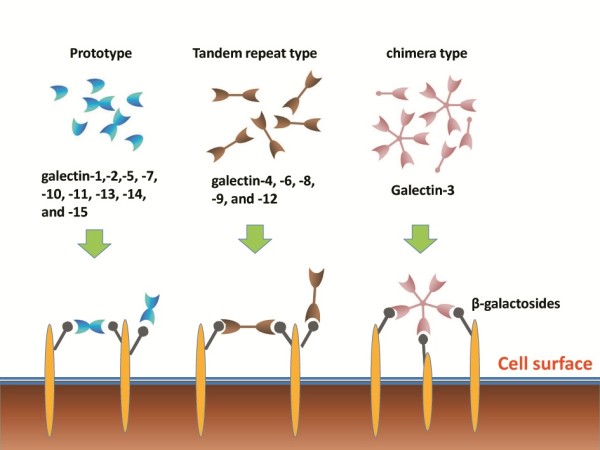
**The structure and classification of different members of the galectin family.** Adapted from [13].

Galectins are a family of evolutionary conserved animal lectins that bind to β-galactosides. They are ubiquitous in mammals and other vertebrate taxa, invertebrates, and fungi [1,2]. First described in the 1970s, galectins are involved in the recognition of carbohydrate ligands during embryogenesis [3]. In recent years, galectins have been shown to have significant immunoregulatory activities, such as cell differentiation, tissue organization, and the regulation of immune homeostasis [4,5]. Galectins have been shown to bind glycans on the surface of bacteria, viruses, protozoa and fungi, which indicates a potential role in the recognition of pathogens [6,7]. So far, 15 galectin members have been identified in a wide variety of tissues [4,8]. All galectins share close sequence homology in their carbohydrate recognition domain (CRD) but exhibit different affinities for different saccharide ligands [9]. Galectins can be bi- or multi-valent in terms of their ligand-binding activity (Figure 1), which accounts for their ability to cross-link cell surface glycoproteins. Based on structural differences, the galectins can be classified into three distinct subgroups [Figure 1]. Prototypic galectins (galectin-1,-2,-5, -7, -10, -11, -13, -14, and −15) have one CRD and are capable of homodimerization. Tandem repeat type galectins (galectin-4, -6, -8, -9, and −12) consist of two distinct CRDs which are joined by a linker of up to 70 amino acids and have differential affinity for carbohydrates. Gal-3 is a unique member of chimera type galectins and exhibits both extracellular and intracellular functions. The protein contains a single CRD with an extended N-terminus which plays a role in protein oligomerization and may participate in the interaction with other intracellular proteins [4,10-12]. This means that Gal-3 can interact with both carbohydrates and proteins.

Gal-3 was first discovered as an IgE-binding protein, and characterized as a 32 kDa antigen (Mac-2) on the surface of murine macrophages [14]. Gal-3 is widely distributed and localizes to the extracellular space, cytoplasmic or nuclear regions. Like other members of the galectin family, Gal-3 does not possess a secretion signal peptide that would direct transport through the classical endoplasmic reticulum-Golgi apparatus secretory pathway [10]. At low concentrations, Gal-3 is a monomer, or can potentially form oligomers but only upon binding to multivalent saccharides, a feature that imparts great flexibility on Gal-3. As a result of the activation of signaling pathways involved in the modulation of a number of cellular processes, Gal-3 can crosslink cell surface receptors, leading to the formation of lattices that cluster these ligands into lipid raft micro-domains [15-18]. These cellular processes include cell-cell adhesion, proliferation, differentiation, apoptosis, and cytokine secretion. Through protein-protein interactions, Gal-3 can react with many extracellular and/or intracellular proteins. This can be done in a carbohydrate dependent or independent manner respectively [19-23].

In recent decades, the literature on Gal-3 has been rapidly growing. The reason generating this increasing interest relates to the broad range of functions displayed by Gal-3. It has now been found that Gal-3 is related to the physiopathology of multiple diseases (Table 1). Gal-3 has been known to be involved in many aspects in asthma, such as eosinophil recruitment [24,25], airway remodeling, development of a Th2 phenotype as well as increased expression of inflammatory mediators [26]. This review will focus on what is known about the Gal-3 and its role in the pathophysiological mechanisms in asthma.

**Table 1:** The levels of serum Gal-3 in different patients

**Disease**	**Method and assay**	**Level in blood (ng/ml)**	**Comments**
Heart failure	ELISA (BG Medicine, Waltham, USA).	Decrease in left ventricular systolic function (LVEDV): 14.7(12.8-18.2); stable LVEDV: 17.9(13.7-22); increase in LVEDV: 19(14.9-24.4).	Patients were divided into three groups according to the change in LVEDV: decrease in LVEDV > 8%, stable LVEDV (−8–8%) and an increase in LVEDV > 8%. Plasma Gal-3 is associated with left ventricular remodeling determined by serial echocardiography and predicts long-term mortality in patients with severe chronic heart failure [27].
Rheumatoid arthritis (RA)	ELISA (R&D Systems, Minneapolis, USA).	Gal-3 was elevated in RA serum and synovial fluids. In RA, serum Gal-3 correlated with C-reactive protein levels.	Gal-3 is not only involved in inflammation, but also contributes to the activation of synovial fibroblasts [28].
Juvenile idiopathic arthritis (JIA)	ELISA (R&D Systems, Minneapolis, USA).	Healthy controls: 8.1(4.9–16.7); inactive disease: 18.6(9.7–28.8); active disease: 35.8(15.8–60.8).	Serum levels of Gal-3 are highest in active JIA children, followed by inactive disease and controls [29].
Behçet’s disease (BD)	ELISA (R&D Systems, Minneapolis, USA).	Active BD patients: 13.08 ± 1.53; inactive BD patients: 8.08 ± 0.71; healthy controls: 7.59 ± 0.48.	Active BD patients had significantly higher levels of serum Gal-3 than inactive patients and controls [30].
Cancer	In house ELISA	Healthy control: 62(20–313); Breast cancer: 100(20–620); Gastrointestinal cancer: 185(20–950); Lung cancer: 171(20–807).	Serum Gal-3 levels were significantly higher in subpopulations of patients having each type of tumor [31].
	ELISA (Bender MedSystems, Vienna, Austria).	Controls: 3.07 ± 0.69; Colorectal cancer: 6.81 ± 4.07	Gal-3 ranged higher in cancer patients than in controls [32].
	ELISA (Human Gal-3 Assay Kit, IBL)	Bladder cancer: 1.07(0.55-2.03) Control: 0.58(0.26-1.26)	Serum Gal-3 concentration of the bladder cancer patients was higher than that of controls [33].
Asthma	ELISA (R&D Systems, Minneapolis, USA).	Asthma serum: 1.5(1.1-1.9); healthy control: 1.8(0.8-2.1).	Gal-3 in asthma serum was lower than that of controls (not published).

Data are expressed as mean ± SD or median (IQR).

### Gal-3 in inflammation

Asthma is a chronic inflammatory respiratory disease characterized by airway inflammation, airway hyperresponsiveness (AHR) and reversible airway obstruction [34]. Treatments targeting eosinophilic inflammation in asthma are able to reduce asthma exacerbations, however the inflammatory mechanisms leading to asthma symptoms and AHR in the absence of sputum eosinophilia is poorly understood. Gal-3 is potentially relevant in the pathogenesis of inflammation in asthma and its phenotypes.

### Gal-3 expression in the inflammatory setting

A variety of tissues and cell types express Gal-3 under basal conditions, including epithelial cells, dendritic cells, macrophages and neutrophils [11,35,36]. However, the pattern of expression can be modulated in the inflammatory setting. *In vivo*, an increase in the extracellular concentration of Gal-3 has been measured in the inflammatory setting in animal models. Bronchoalveolar lavage (BAL) fluid from ovalbumin (OVA) challenged mice contained significantly higher levels of Gal-3 compared to control mice [24], similar to the results from mice infected with *Streptococcus* pneumonia [37]. Elevated levels of Gal-3 were also detected in prion-infected brain tissue [38], and in synovial tissue and serum from patients with rheumatoid arthritis (RA) [28]. In RA, serum Gal-3 levels were increased further in uncontrolled disease. In human asthma, highly variable Gal-3 expression was detected on both sputum macrophages and neutrophils by flow cytometry, and although it tended to be lower in asthmatic patients compared to healthy controls, this difference did not reach statistical significance [39].

Similarly, both intracellular and surface expression of Gal-3 are enhanced after several different stimuli. Increased Gal-3 protein was detected in muscle endothelium by immunohistology accompanied by elevated Gal-3 in the serum of mice fed with a diet containing 60% fat calories [40]. Elevated levels of Gal-3 were also measured in both alveolar vascular endothelial cells and alveolar macrophages, indicating both cell types as a potential source of the elevated Gal-3 [41]. In human endothelium, Gal-3 is regulated at the protein level in response to IL-1β, and at the mRNA level in response to advanced glycation end products casein (AGE-Cas) [42]. These findings are consistent with upregulation of Gal-3 with immune activation, since dietary fat and IL-1β are involved in innate immune activation. Furthermore, macrophages in the BAL of OVA challenged mice expressed large amounts of Gal-3, and these were the major cell type that contained Gal-3 [24]. In addition, the increased level of Gal-3 has also been detected on the surface of neutrophils [43], eosinophils [44], mast cell, monocytes and lymphocytes [25].

### Regulation of leukocyte trafficking and activation

**Figure 2:**
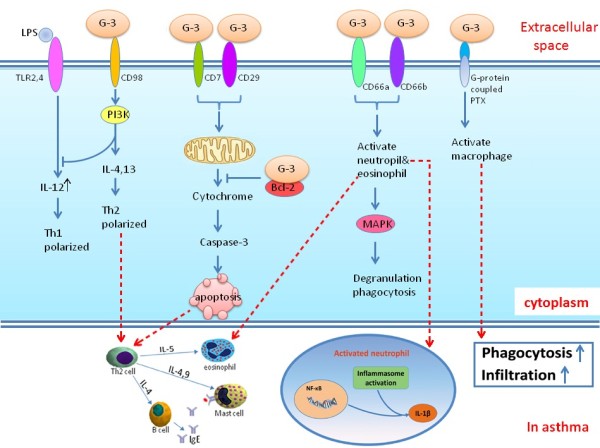
**The intracellular and extracellular functions of galectin-3.** The blue arrow indicates positive effects, the T-shaped end indicates negative effects. LPS, lipopolysaccharide; TLR, Toll-like receptor; IL, interleukin; Th, helper T cell; PI3K, phosphatidylinositol 3-kinase; G-3, galectin-3; PTX, Pertussis toxin; BCL-2, B-cell lymphoma-2; MAPK, Mitogen-activated protein kinase. Adapted from [26,51-53].

An increasing number of studies has demonstrated that Gal-3 plays a critical role in the process of leukocyte trafficking, activation and cytokine release. One facet of inflammation where Gal-3 appears to have beneficial effects is phagocytosis, which is necessary to clear pathogens, foreign bodies and cellular debris, thus allowing inflammation to resolve. Gal-3 can also regulate cell apoptosis from both inside and outside the cell (Figure 2) [45,46]. Moreover, Gal-3 is a unique member of the family with both anti- and pro-apoptotic activity [47]. Cytoplasmic Gal-3 binding to Fas would inhibit apoptosis by localising to the mitochondrial membrane to maintain mitochondrial membrane integrity and preventing the cytochrome c release [45,48-50]. In contrast, extracellular Gal-3 directly induces T cell death in a carbohydrate-dependent manner by binding to its cell surface receptors, such as CD7, CD29 [46].

### Macrophage/monocyte

Gal-3, as a chemoattractant and adhesion factor, plays an important role in the trafficking of monocytes and macrophages. *In vitro*, recombinant human Gal-3 induces monocyte/macrophage migration. These processes could be inhibited by addition of lactose, specific mAb, and the C-terminal domain fragment. In addition, Gal-3 causes Ca^2+^ influx in monocytes, and both the chemotactic effect and the induction of Ca^2+^ influx involve a pertussis toxin-sensitive pathway, which suggests a role for G protein-coupled receptors [52]. *In vivo*, Gal-3-deficient mice develop significantly reduced numbers of peritoneal macrophages and granulocytes compared with wild-type mice when treated with thioglycolate i.p. [54,55].

Gal-3 can activate monocytes/macrophages through its lectin function (Figure 2). *In vitro*, Gal-3 (ie, approximately 10–100 nM) can induce optimal superoxide release from monocytes [52,56] and promote the uptake of apoptotic neutrophils from monocyte derived macrophages [57]. Conversely, Gal-3 deficient macrophages exhibited reduced phagocytosis of IgG-opsonized erythrocytes and apoptotic thymocytes *in vitro* compared to wild type cells. In addition, Gal-3−/− mice showed attenuated phagocytic clearance of apoptotic thymocytes by peritoneal macrophages *in vivo*. These mice also exhibited reduced IgG-mediated phagocytosis of erythrocytes by Kupffer cells in a murine model of autoimmune hemolytic anemia [58]. This is further corroborated by *in vitro* studies in which Gal-3 null macrophages demonstrate reduced phagocytosis of apoptotic neutrophils [37].

Alternative macrophage activation has been implicated in asthma [59-61]. Gal-3 has a property of negative regulation of LPS function, which protects the host from endotoxin shock while increasing *Salmonella* survival. In contrast, blocking Gal-3 binding sites enhanced LPS-induced inflammatory cytokine expression by wild-type macrophages [62]. Furthermore, Gal-3 deficient mice infected with *Toxoplasma gondii*, produced larger amounts of IL-12, and induced Th1 polarized immune response (Figure 2) [63]. The disruption of the Gal-3 gene specifically restrains IL-4/13-induced alternative macrophage activation without affecting IFN-γ/LPS-induced classical activation or IL-10-induced deactivation. This results were supported by other recent studies [64,65].

### Neutrophil

Gal-3 promotes the adhesion of human neutrophils [66,67]. Furthermore, in an *in vivo* streptococcal pneumonia mouse model, neutrophil extravasation was closely related to accumulation of Gal-3 in the alveolar space, which was β_2_-integrin independent [67]. In peripheral blood neutrophils, cross-linking of CD66b, a candidate receptor for Gal-3, mediates the release of interleukin-8 from intracellular storage [68], the most potent chemoattractant for neutrophils. Some other results, in line with a decreased cellular infiltrate observed in numerous *in vivo* models of inflammation performed in Gal-3 knockout mice, have provided more evidence for a role for this protein in mediating leukocyte recruitment during an inflammatory response [41,55,63,69]. One of the possible explanations of the trafficking mechanisms is that the cross-linking of neutrophil CD66a and/or CD66b, the functional Gal-3 receptors, resulted in increased adhesion of the neutrophils to endothelial cells [68,70]. This hypothesis has been confirmed by the observation through confocal microscopy recently [71].

Concomitantly, Gal-3 can also activate neutrophils and enhance their phagocytic capabilities. Recombinant human Gal-3 could enhance human neutrophils to release superoxide through recognition of special cell surface glycoproteins. This activation is dose-dependent and the lectin property of Gal-3 is intrinsic to its carboxyl-terminal domain. Lactose could inhibit this process [72]. In addition, Gal-3 can also increase L-selectin shedding and interleukin-8 production in naïve and primed neutrophils. These activities required the presence of the C-terminal lectin domain and the N-terminal nonlectin domain of Gal-3. On the other hand, after Gal-3 binds to primed neutrophils, the cells can cleave Gal-3, mainly through elastase, which damages the N-terminal domain of Gal-3 [73].

*In vivo* Gal-3−/− mice develop more severe pneumonia after infection with *S. pneumoniae*, as demonstrated by increased bacteremia and lung damage compared to wild-type mice. Gal-3 reduces the severity of *pneumococcal* pneumonia in part by augmenting neutrophil phagocytosis of bacteria and delaying neutrophil apoptosis [37]. The mechanism of the increased phagocytosis of neutrophils by Gal-3 may be through the MAPK pathway and CD66 surface expression [Figure 2]. Disruption of this signaling pathway abrogated Gal-3 mediated modulation of neutrophil degranulation and phagocytosis [70,74].

### Eosinophils

*In vitro*, recombinant human Gal-3 can directly increase rolling and adhesion of eosinophils from allergic donors in an α-4 integrin-dependent manner, with an effect comparable to that evoked by vascular cell adhesion molecule (VCAM)-1. These activities could be inhibited by specific Gal-3 mAbs as well as lactose [44]. Furthermore, CD66b, as an activation marker for human granulocytes, engaged by mAb or Gal-3, activated a Src kinase family molecule and resulted in cellular adhesion, superoxide production, and degranulation of eosinophils. Disruption of CD66b inhibited the adhesion and activation of eosinophils [53]. *In vivo* studies in Gal-3 knockout mice exhibited significantly lower eosinophil infiltration, serum IgE and IL-4 (Th2 cytokine) levels compared with wild type counterparts. This may indicate a direct effect for Gal-3 on eosinophil trafficking or suggest that Gal-3 is critical for the development of inflammatory Th2 responses. In its absence, mice develop a Th1-polarized response [25].

In contrast to these experiments, intratracheal instillation of plasmid DNA encoding Gal-3 in a OVA challenged-rat model led to normalization of the eosinophil and T cell count in BALF and that there was a strong concomitant inhibition of IL-5 mRNA in the lungs [75]. Twelve weeks after the first intranasal antigen instillation in chronically asthmatic mice, treatment with the Gal-3 gene led to an improvement in the eosinophil count and the normalization of hyperresponsiveness to methacholine. Concomitantly, this treatment resulted in an improvement in mucus secretion and subepithelial fibrosis in the chronically asthmatic mice, with a quantitatively measured reduction in lung collagen, a prominent feature of airway remodeling [76]. Similarly, treatment of chronic asthmatic mice with gene therapy using plasmid encoding Gal-3 led to inhibition of suppressor of cytokine signaling (SOCS) proteins 1 and 3, which led to an improvement in Th2 allergic inflammation [77]. Therefore, these results indicate that treatment with a plasmid encoding Gal-3 may not exactly reproduce the function of endogenous Gal-3, possibly because the protein may be expressed differently in the cells or tissues, in the intra- versus extra-cellular modes of action, and in monomer or polymer between mice expressing a transgene and wild-type mice.

### Other cells

Gal-3 also regulates the migratory pattern of dendritic cells (DCs). Gal-3 deficient DCs exhibited defective chemotaxis. Moreover, exogenous Gal-3 displays the activation of mast cells, such as mediator release [78,79], and the increased apoptosis of mast cells [80]. Gal-3 deficient mast cells showed a significantly lower amount of histamine, the cytokine IL-4, expression of IgE receptor and passive cutaneous anaphylaxis reactions [81].

In T cells, Gal-3 inhibits apoptosis by interacting with Bc1-2 in a lactose-inhibitable manner [45], and is necessary for IL-2 dependent cell growth [82]. Conversely, extracellular Gal-3 directly induces death of human thymocytes and T cells by binding to T cell surface glycoprotein receptors, such as CD7, CD29, CD43, CD45 and CD71 [83-88].

### Gal-3 in experimental models of asthma

In a murine model of asthma treated with OVA, Gal-3+/+ mice developed significantly enhanced allergic airway inflammation and AHR. Firstly, Gal-3 expression was significantly elevated in the airways of Gal-3+/+ mice, not only in the peribronchial inflammatory cells, but also in the fluid lining the airways as well. Secondly, Gal-3+/+ mice exhibit significantly elevated allergic airway inflammation, with an increased number of eosinophils compared with similarly treated Gal-3−/−. Thirdly, Gal-3−/− mice exhibited lower goblet cell metaplasia compared to Gal-3+/+ mice. Fourthly, Gal-3+/+ mice exhibited higher serum IgE levels than similarly treated Gal-3−/− mice. Fifthly, Gal-3 null mice display a lower Th2 response but a higher Th1 response. Finally, Gal-3+/+ mice manifest significantly higher airway responsiveness to methacholine compared to Gal-3−/− mice [24,89]. Furthermore, bone marrow derived mast cells (BMMC) from Gal-3 deficient mice not only secreted significantly lower levels of histamine and IL-4, but also exhibited lower expression of IgE receptor and reduced passive cutaneous anaphylaxis reactions compared with Gal-3+/+ BMMC. In addition, Gal-3−/− BMMC contained a significantly lower basal level of JNK1 protein than Gal-3+/+ BMMC, which is probably responsible for the lower IL-4 expression [81]. In a mouse model of chronic allergic airway inflammation exposed to OVA for 12 weeks, Gal-3−/− mice displayed significantly lower airway inflammatory responses than did wild-type mice, and lower amounts of airway remodeling [26].

### Gal-3 in human asthma

The inflammatory response in asthma shows heterogeneity, which involves many cells and cellular elements [90]. Recognizing the different inflammatory phenotypes within asthma is important for understanding the underlying disease processes. The different inflammatory phenotypes are also clinically relevant due to potentially differing responses to therapeutic interventions. An important classification of asthma has been performed by Simpson JL, *et al.*, in which asthmatic subjects were classified into four groups based on the presence of neutrophils and eosinophils using the 95th percentile from the healthy control subjects as a cut-off point [91]. This resulted in four inflammatory subtypes, including neutrophilic asthma, eosinophilic asthma, mixed granulocytic asthma and paucigranulocytic asthma. Recent years, many studies have demonstrated the distinct mechanisms of these subgroups, which are important because each subtype has a distinct mechanism and differential responses to therapy [92-98]. The mechanisms of eosinophilic asthma involve activation of Th2 pathways, typically by allergen, and release of Th2 cytokines, such as IL-4, 5, 9 and 13. Bronchial biopsies from these patients show infiltration with eosinophils, activated mast cells, and T cells that are predominantly Th2 cells [99].

However, the mechanisms of non-eosinophilic asthma are different from that of eosinophilic asthma. The neutrophilic form of asthma appears to be driven by infection and pollutant activation of innate immune responses, leading to active IL-1β secretion via TLR and NLRP3 inflammasome activation [100,101].

It is likely that Gal-3 might be important in non-eosinophilic forms of asthma. In murine models, exogenous Gal-3 has been linked to more severe AHR [24,26], but this effect is associated with down regulation of IL-5 gene expression after the treatment with plasmid encoding Gal-3 [75,76] and therefore the presence of non-eosinophilic airways inflammation. While it is unknown if these kinds of experiments represent the function of endogenous Gal-3 these initially paradoxical effects (more AHR and less Th2 inflammation) can be explained in the context of an inflammatory phenotype, which displays the suppressed Th2 cytokines, and persistent AHR indicates a non-eosinophilic phenotype. Consistent with this, Gal-3 is present on sputum macrophages and neutrophils in asthma [39]. So far, studies of the level of Gal-3 in human asthma have not analyzed data by inflammatory phenotype. Available data only shows reduced Gal-3 gene expression in asthmatic sputum cells [39]. So, there is a need to elucidate how Gal-3 is involved in the mechanisms of asthma phenotypes.

## Conclusion

As a multifunctional protein widely expressed by many types of inflammatory cells, Gal-3 overexpression and change of inter- and sub-cellular localization are commonly seen in various types of inflammatory cells. Growing evidence has shown that Gal-3, first discovered as an IgE-binding protein, is an important regulator of inflammatory cell infiltration, activation, and clearance. Recent studies of the murine models using Gal-3 gene transfer indicate that Gal-3 is anti-inflammatory, however these results might not represent the effect of endogenous Gal-3. In fact, a large number of in *in vivo* and *in vitro* studies suggest the Gal-3 is pro-inflammatory. This perplexing paradox may be explained by considering the heterogeneity of airway inflammation in asthma and the specific effects of Gal-3 as a mechanism of noneosinophilic forms of asthma. At present there are limited data available for levels and function of Gal-3 in human asthma or chronic obstructive pulmonary disease. Therefore, targeting the actions of Gal-3 might elucidate underlying molecular mechanisms of asthma and represent a promising therapeutic strategy for the development of effective therapeutic agents for asthma treatment.

## Abbreviations

AHR: Airway hyperresponsiveness; BALF: Bronchoalveolar lavage fluid; BCL-2: B-cell lymphoma-2; BMMC: Bone marrow derived mast cells; CRD: Carbohydrate recognition domain; Gal-3: Galectin-3; IL: Interleukin; LPS: Lipopolysaccharide; MAPK: Mitogen-activated protein kinase; OVA: Ovalbumin; PI3K: Phosphatidylinositol 3-kinase; PTX: Pertussis toxin; RA: Rheumatoid arthritis; SOCS: Suppressor of cytokine signaling; TLR: Toll-like receptor; Th: Helper T cell.

## Competing interests

The authors declare that they have no competing interests.

## Authors’ contributions

PG drafted the manuscript. JS reviewed and revised it critically for important intellectual content. JZ and PGG have made substantial contributions to conception and design, and have given final approval of the version to be published. All authors read and approved the final manuscript.

## Authors’ information

J Zhang and PG Gibson are joint corresponding authors to this manuscript.
